# FoxQ1 Promotes Glioma Cells Proliferation and Migration by Regulating NRXN3 Expression

**DOI:** 10.1371/journal.pone.0055693

**Published:** 2013-01-30

**Authors:** Hong-Tao Sun, Shi-Xiang Cheng, Yue Tu, Xiao-Hong Li, Sai Zhang

**Affiliations:** Institute of Traumatic Brain Injury and Neurology of the Chinese People's Armed Police Forces, Tianjin, China; Beijing Tiantan Hospital, Capital Medical University, China

## Abstract

**Background:**

Forkhead box Q1 (FoxQ1) is a member of the forkhead transcription factor family, and it has recently been found to participate in cancer development. However, whether FoxQ1 expression contributes to glioma development and progression is not known. We investigate FoxQ1 expression in gliomas and the role of FoxQ1 during tumorgenesis.

**Methods:**

Reverse transcription quantitative real-time PCR (RT-qPCR) and Western blot were used to determine the FoxQ1 and Neurexins 3 (NRXN3) expression in gliomas. Chromatin immunoprecipitation (ChIP) and luciferase assays were used to determine the regulation between FoxQ1 and NRXN3. We established depleted FoxQ1 stable clones in U-87MG cells and overexpressed FoxQ1 stable clones in SW1088 cells. MTT and transwell were used to evaluate the ability of proliferation and migration, respectively.

**Results:**

FoxQ1 mRNA and protein were up-regulated in gliomas and negatively related to the NRXN3 expression (*r* = −0.373, *P* = 0.042). FoxQ1 directly binds to NRXN3 promoter region and suppresses the promoter activity. Furthermore, the ability of proliferation and migration is reduced in depleted FoxQ1 cells.

**Conclusion:**

FoxQ1 promotes glioma cell proliferation and migration by down-regulation of NRXN3 expression.

## Introduction

Gliomas are the most common tumors of the central nervous system in adults. Patients with glioblastoma routinely undergo surgery followed by adjuvant radiation therapy and chemotherapy. Although diagnosis and therapeutic strategies have been progressed, the median survival times still less than 1 year in most cases. The 5-year survival rate in patients with glioma is among the lowest for all cancers [1], [2]. The understandings of the biology and molecular mechanisms of glioma development and progression are not well known.

The forkhead box (Fox) gene family is a large and diverse group of transcription factor and plays important roles in biological processes, including development, metabolism, immunology, and senescence [3], [4]. Loss or gain of Fox function can alter cell fate and promote tumorigenesis as well as cancer progression [5]. Thus, Fox proteins are potential targets for therapeutic intervention, as well as biomarkers for predicting and monitoring treatment responses.

Forkhead box Q1 (FoxQ1, also known as HFH1) is a member of the Fox transcription factor family, which is a predominant regulator of the cell cycle [6]–[9]. The biological function of FoxQ1 has been clearly identified in hair follicle differentiation [10], [11]. Previous studies have found that FoxQ1 is widely expressed at the mRNA level in murine tissues, with particularly high expression levels in the bladder and stomach [12]. Recent studies have been reported that FoxQ1 is markedly overexpressed in colorectal cancer and enhances tumorigenicity and tumor growth *in vivo* [13]. Furthermore, FoxQ1 is also involved in epithelial-mesenchymal transition regulation by suppressing E-cadherin transcription and is associated with aggressive cancer phenotype [14], [15]. However, whether FoxQ1 expression contributes to glioma development and progression is not known.

Neurexins belong to a family of highly polymorphic neuronal-specific cell surface proteins, whose structure suggests a role in cell adhesion and recognition [16]–[18]. The three neurexin genes, NRXN1 (2q16.3), NRXN2 (11q13.2) and NRXN3 (14q31.1), each display two promoters for a longer transcript, α-neurexins and a shorter transcript, β-neurexins were identified [19]. Differential promoters produce six primary transcripts, which are then subjected to alternative splicing at five positions [19], [20]. Several of these isoforms bind to ligands that include membrane-bound endogenous neuroligins and dystroglycans, soluble endogenous neurexophilins and a-latrotoxin spider neurotoxins [10], [18], [21]. Polymorphic site of NRXN3 gene (rs10146997) was significantly associated with higher risk of breast cancer development [22]. In addition, NRXN3 polymorphisms are associated with alcohol dependence [23]. However, no data regarding cellular and biological functions of human NRXN3, especially in cancer cells, are available.

In the present study, we showed that FoxQ1 expression was higher in glioma specimens than the normal tissues, whereas NRXN3 expression was lower in glioma specimens. Furthermore, FoxQ1 expression negatively related to NRXN3 expression in human glioma tissues. Thus, we hypothesized that FoxQ1 promotes glioma development by downregulation of NRXN3 expression.

## Materials and Methods

### Cell culture and human samples

The human glioma cell line Hs683, SW1088, LN-229, and U-87MG were purchased from American Type culture Collection (ATCC) and maintained in DMEM (Gibco) supplemented with 10% fetal bovine serum (FBS, Gibco) and 1% penicillin/streptomycin in a humidified atmosphere of 5% CO_2_ at 37 °C. Normal human astrocytes (NHA) were obtained from Gibco human astrocytes kit and cultured in astrocyte medium (Gibco).

Glioma specimens were obtained from Affiliated Hospital of Logistics College of CPAPF. This study was approved by the Institutional Review Board of the Affiliated Hospital of Logistics College of CPAPF and written consent was obtained from all participants. All tumors were from patients with a newly diagnosed glioma who had received no therapy before sample collection. After radical prostatectomy, tissues were flash-frozen in liquid nitrogen and stored at −80 °C.

### Reverse transcription quantitative real-time PCR (RT-qPCR)

Total RNA was extracted with TRIZOL reagent according to the manufacturer's instructions. 5 µg of total RNA was used to perform reverse transcribed by using SuperScript II and oligo dT following the manufacturer recommendations (Invitrogen). The RT-qPCR analysis was performed using the Fast SYBR Green MasterMix System (Invitrogen) according to the manufacturer's instructions. The targeted gene relative quantification was given by the CT values, and the CT value of GAPDH was subtracted to obtain ΔCT. The relative mRNA expression level of targeted genes was determined as 2^−ΔCT^. The experiment was performed in triplicate.

### Western blot

A quantity of 30 µg of whole cell lysates per sample was separated by SDS-PAGE using 10% polyacrylamide gels and transferred to PVDF membrane which was subsequently incubated with polyclonal rabbit anti-FoxQ1, anti-NRXN3 (Abcam) and a second antibody (anti-rabbit IgG, Santa Cruz Biotechnology). The same membranes were stripped and blotted with an anti-β-actin antibody (Sigma) and used as loading controls. The probe proteins were detected using the Amersham enhanced chemiluminescence system according to the instructions of the manufacturer.

### Plasmids and stable transfection of glioma cells

The cDNA fragment encoding human full length FOXQ1 was isolated using reverse transcriptase-polymerase chain reaction (RT-PCR) using total RNA from U-87MG cell line. The primers sequences were as following: FoxQ1 (forward, 5′- GGAATTCATGAAGTTGGAGGTGTTCGTC-3′ and reverse, 5′- CCTCGAGCGCTACTCAGGCTAGGAGCGTCTCCAC-3′). The PCR product was cloned into EcoR I and Xho I sites of the mammalian expression vector pcDNA3.1 (+) (Invitrogen). The FoxQ1 and NRXN3 shRNA plasmids were purchased from Santa-Cruz biotechnology. Stably transfected cell lines were isolated by neomycin (G418) selection.

### Promoter reporters and luciferase assay

The NRXN3 promoter (−1500∼+1) was amplified by from genomic DNA of U-87MG cells and the fragment was cloned into the Bgl II and Kpn I restriction sites in the luciferase reporter plasmids pGL3-basic vector (Promega) (pGL3-NRXN3). We generated mutant NRXN3 promoters by Fast Mutagenesis System (TransGen Biotech). For luciferase assay, 5×10^4^ cells per well in 12-well plates were cultured without antibiotics overnight and then transfected with NRXN3 promoter reporter plasmids. After 24 hours, cells were washed with phosphate-buffered saline (PBS), subjected to lysis, and their luciferase activities measured by using a dual luciferase assay kit (Promega). The results were normalized against Renella luciferase. All transfections were performed in triplicate.

### Chromatin immunoprecipitation assay

ChIP was carried out using kit from Upstate Biotechnology according to manufacturer's protocol. Briefly, U-87MG cells were transfected with pcDNA3.1-FoxQ1 or vector control. The putative binding sites of NRXN3 were amplified with the following primers: site 1, 5′- ATTCCTTCTAAGACTTTGGAG-3′ and 5′-GGTGATGTTAGAGATACTAGG-3′; site 2, 5′- GCAGAGGAGTAAAGTGGAAT-3′ and 5′- AGAAATGAGCACAGGTGATG-3′. The PCR products were resolved electrophoretically on a 2% agarose gel and visualized by ethidium bromide staining.

### MTT assay

For MTT assays, 2×10^3^ cells in 200 µl culture medium were plated into a well of 96-well plates. After culturing cell for an appropriate time, 10 µl of 5 mg/ml MTT was added into each well and cultured for 4 h. Then, the cell culture medium was replaced by 100 µl of dimethyl sulfoxide. Thirty minutes after dimethyl sulfoxide addition, the plates were placed on a microplate autoreader (Thermo). Optical density was read at 570 nm wavelength and cell growth curves were determined according to the optical density value.

### Transwell assay

For transwell assay, 1×104 cells were cultured in the upper chamber with serum-free medium. The lower chamber contained complete medium (10% fetal bovine serum). After incubation for 12 hours, cells adherent to top surface of the membrane were removed with a cotton applicator, whereas cells migrated to bottom surface were fixed with 70% methanol and stained with crystal violet. The migrated cells on the bottom surface of the membrane were photographed and counted on an inverted microscope.

### Xenograft assay

Glioma cells (1×10^6^) were injected intracranially into nude mice (n = 5, per group). Mice were euthanized when they were moribund or on day 90 after glioma cell injection.

### Statistical analysis

Results of *in vitro* experiments were depicted as mean ±SD and student's t-test (two-tailed) was used to compare values of test and control samples. All calculations were performed with the SPSS for Windows statistical software package (SPSS Inc). The level of significance was set to *P*<0.05.

## Results

### FoxQ1 is overexpressed in human glioblastomas and negatively correlates with NRXN3 expression

**Figure 1:**
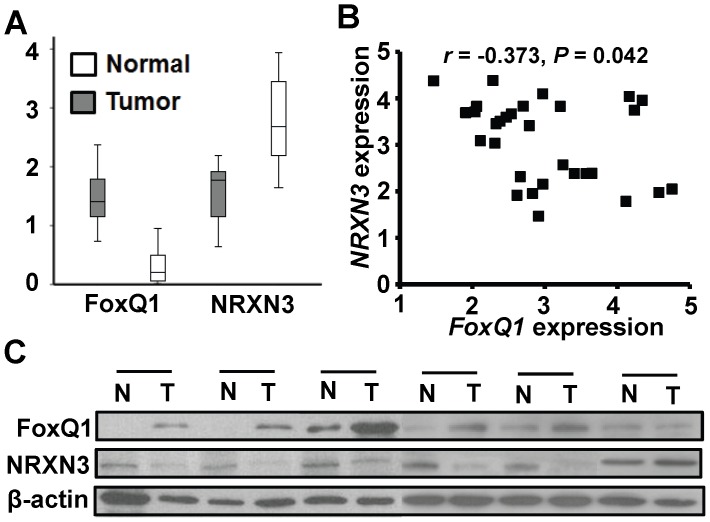
Expression of FoxQ1 and NRXN3 in human normal brain and glioma tissues. **A**, *NRXN3* and *FoxQ1* mRNA expression by RT-qPCR. The mRNA expression was analyzed in 30 matched primary glioblastoma tissues and the adjacent normal brain tissues. **B**, FoxQ1 expression levels correlated negatively with NRXN3 expression levels in glioblastoma samples (Pearson's correlation test *r* = −0.373; *P* = 0.042). **C,** Expression of FoxQ1 and NRXN3 protein in primary glioblastoma tissues and the adjacent normal brain tissues. Normal (N) and tumor (T) samples were analyzed by western blot. β-actin used as the loading control.

We first determined the *FoxQ1* and *NRXN3* mRNA expression in 30 human glioblastoma and the paired adjacent normal brain specimens by RT-qPCR analyses. The results indicated *FoxQ1* mRNA expression was up-regulated in glioma specimens. Furthermore, we observed for *NRXN3* mRNA expression, we found that *NRXN3* mRNA was down-regulated in tumor cells than the paired adjacent normal brain tissues (Fig. 1A). In addition, we found a significant correlation between the *FoxQ1* and *NRXN3* mRNA expression levels (Fig. 1B; *r* = −0.373, *P* = 0.042). Furthermore, we performed Western blot analyses using total protein extracts in 4 matched human glioblastoma (T) and adjacent normal tissues (N). As shown in Fig. 1C, FoxQ1 protein levels were upregulated in 5 of 6 malignant tumor samples, whereas NRXN3 protein levels were downregulated in the 5 malignant tumor samples. Furthermore, NRXN3 expression negatively correlated with FoxQ1 expression in 5 of 6 paired samples. Therefore, our results suggest that NRXN3 expression negatively related to FoxQ1 expression in human glioma tissues.

### FoxQ1 suppresses NRXN3 expression in glioma cells

**Figure 2:**
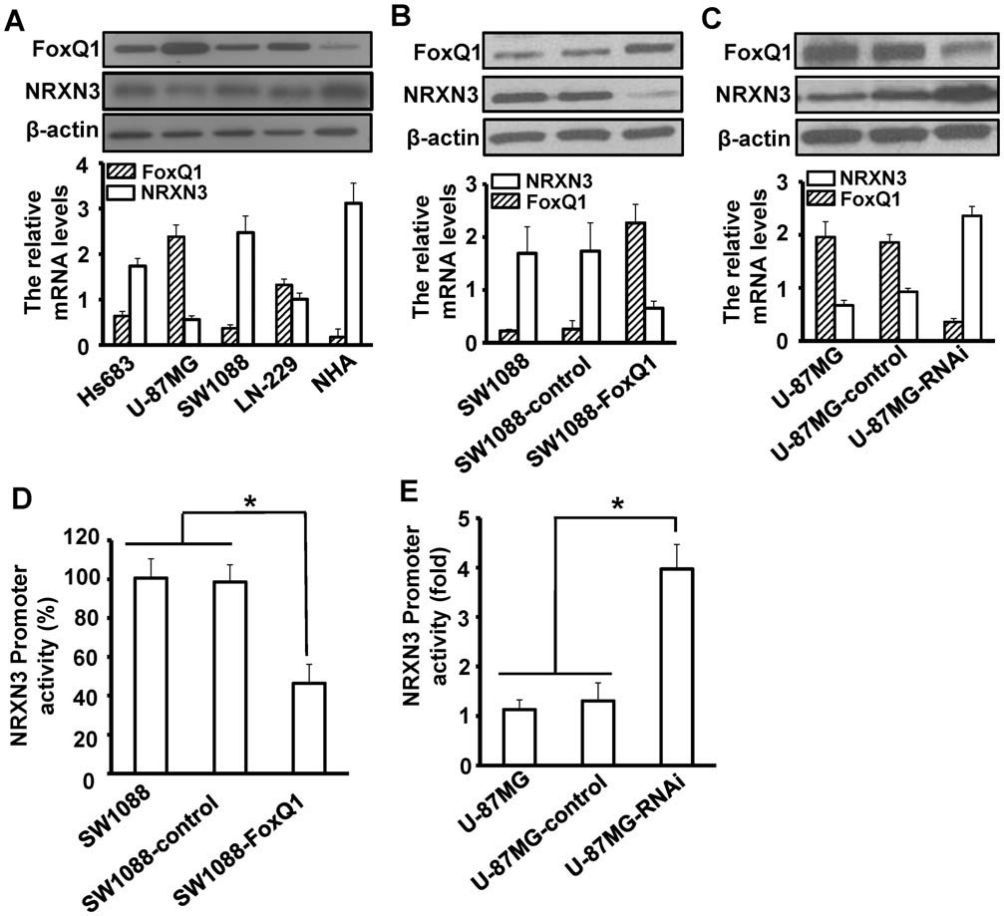
FoxQ1 suppress the NRXN3 expression in human glioma cell lines. **A**, Determination of FoxQ1 and NRXN3 expression in human glioma cell lines and normal human astrocytes using RT-qPCR (lower) and Western blot (upper). **B**, Up-regulation of NRXN3 mRNA and protein expression by overexpressing FoxQ1. FoxQ1 and NRXN3 expression levels in parental, control, SW1088-FoxQ1 cells by RT-qPCR (lower) and Western blot (upper). **C**, Down-regulation of NRXN3 mRNA and protein expression by depletion of FoxQ1 expression. FoxQ1 and NRXN3 expression in parental, control, and U-87MG-RNAi cells by RT-qPCR (lower) and Western blot (upper). **E+F**, Effect of FoxQ1 on NRXN3 promoter activity. Repression of the NRXN3 promoter in SW1088-FoxQ1 cells (E) and transactivation of the NRXN3 promoter in U-87MG-RNAi cells (F). Inhibition was calculated as a percentage relative to U-87MG cells and activation was calculated relative to SW1088 cells. Three independent experiments were conducted.

To determine the FoxQ1 and NRXN3 expression levels in glioma cells lines and normal human astrocytes cells, we examined the FoxQ1 and NRXN3 expression in Hs683, U-87MG, SW1088, LN-229 and NHA cells by RT-qPCR and Western blot (Fig. 2A). The results showed that higher expression of FoxQ1 mRNA and protein was evident in Hs683, U-87MG, SW1088 and LN-229 glioma cells than the normal human astrocytes. Moreover, the FoxQ1 expression levels were negatively related to the NRXN3 levels. To determine the effect of increased FoxQ1 expression on NRXN3 expression, we studies SW1088, which had low levels of the FoxQ1 expression. We transfected these cells with FoxQ1 expression vector pcDNA3.1-FoxQ1 as well as their vector control. We found that the FoxQ1-transfected cells exhibited significantly decreased NRXN3 mRNA and protein expression (Fig. 2B).

Conversely, to determine the effect of decreased FoxQ1 expression on NRXN3 expression, we transfected FoxQ1 shRNA into U-87MG cells, which typically express high levels of FoxQ1 as well as their vector control. The FoxQ1 mRNA and protein expression were significantly decreased in U-87MG-RNAi than the control and parental cells (Fig. 2C); the cells exhibited significantly increased NRXN3 mRNA and protein expression (Fig. 2C). Our results indicate that suppression of FoxQ1 expression increase NRXN3 expression in glioblastoma cells.

### FoxQ1 regulates *NRXN3* promoter activity in glioma cells

To investigate the role of FoxQ1 in regulating NRXN3 transcription, we explored whether FoxQ1 regulates NRXN3 promoter activity. The NRXN3 promoter luciferase construct pGL3-NRXN3 was transfected into SW1088 cells with pcDNA3.1-FoxQ1 or the vector control. The luciferase activity was higher in SW1088-FoxQ1 cells than the control and parental cells (Fig. 2D). Conversely, to estimate the effect of decreased FoxQ1 expression on NRXN3 transcription, we knocked down the FoxQ1 expression by co-transfecting FoxQ1 shRNA and the NRXN3 promoter into U-87MG cells. The luciferase activity was lower in U-87MG-RNAi cells than the control and parental cells (Fig. 2E). These results suggest that FoxQ1 inhibit the NRXN3 promoter activity in glioma cells.

### Direct interaction of FoxQ1 with the NRXN3 promoter

**Figure 3:**
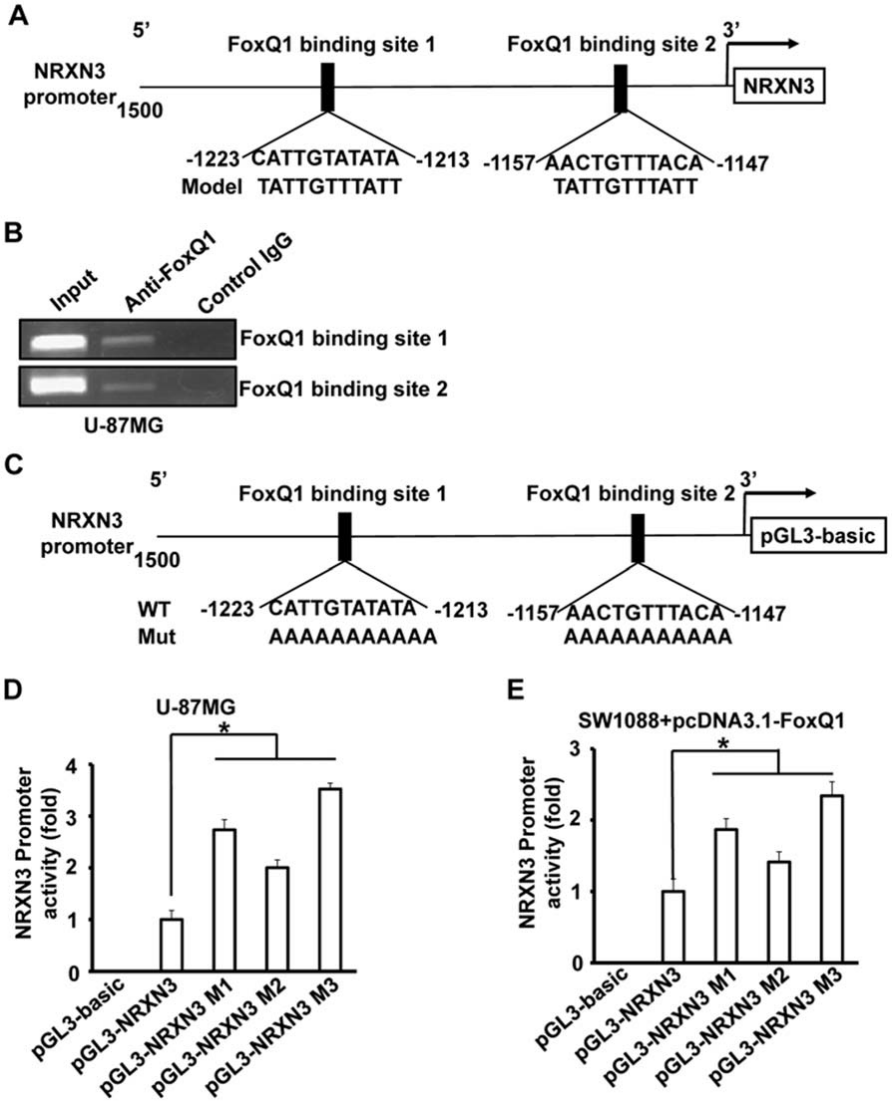
The NRXN3 as a transcriptional target of FoxQ1. **A**, Sequence and position of putative FoxQ1 binding sites on the NRXN3 promoter. **B**, ChIP assays were done with U-87MG cells. Chromatin fragments of the cells were immunoprecipitated with anti-FoxQ1 antibody or negative control IgG (middle) and subjected to PCR. We subjected 1% of the total cell lysates to PCR before immunoprecipitation as inputs. **C**, schematic structure of the NRXN3 promoter. The sequence of the FoxQ1 binding sites are shown in both wild-type (WT) and mutant (Mut) forms. **D+E**, Luciferase activity with or without mutations in NRXN3 promoter. U-87MG cells were transfected with the wild-type NRXN3 promoter or its mutants (D). SW1088 cells were co-transfected with the wild-type NRXN3 promoter or its mutants and pcDNA3.1-FoxQ1 (E). Luciferase activities were then determined. Three independent experiments were conducted. * *P*<0.05

To determine whether NRXN3 could be a direct transcriptional target of FoxQ1, we analyzed the sequence of the NRXN3 promoter by using the MAPPER [24]. We identified two putative FoxQ1 binding sites in the *NRXN3* upstream promoter region (Fig. 3A). To demonstrate that FoxQ1 directly binds to endogenous NRXN3 promoter region, we performed chromatin immunoprecipitation assays with U-87MG cells. We found that endogenous FoxQ1 protein bound to both of the FoxQ1 binding sites of the *NRXN3* promoter (Fig. 3B). Thus, our results indicate that FoxQ1 directly bind to NRXN3 promoter region *in vivo*.

### FoxQ1 binding sites are critical for the suppression of the NRXN3 promoter in glioma cells

To assess the functional role of the FoxQ1 binding sites in NRXN3 regulation, we performed site-specific mutagenesis within the FoxQ1-binding sites of the NRXN3 promoter pGL3-NRXN3. As shown in Fig. 3C, various mutant reporters were generated from the wild-type NRXN3 promoter construct, including a FoxQ1-binding site 1 mutation only (pGL3-NRXN3-Mut1), a FoxQ1-binding site 2 mutation only (pGL3-NRXN3-Mut2), and both site 1 and site 2 mutations (pGL3-NRXN3-Mut3). We transfected these mutant luciferase reporters into U-87MG cells and compared the activity with that of wild-type NRXN3 promoter pGL3-NRXN3. Disruption of one or both of the FoxQ1-binding sites significantly increased NRXN3 promoter activity (Fig. 3D). In addition, disruption of one or both of the FoxQ1 binding sites significantly inhibited NRXN3 promoter repression by pcDNA3.1-FoxQ1 in SW1088 cells (Fig. 3E). These results suggest that the FoxQ1 binding site is critical for the NRXN3 promoter suppression in glioma cells.

### Altered FoxQ1 expression affects the proliferation and migration of glioblastoma cells *in vitro*

**Figure 4:**
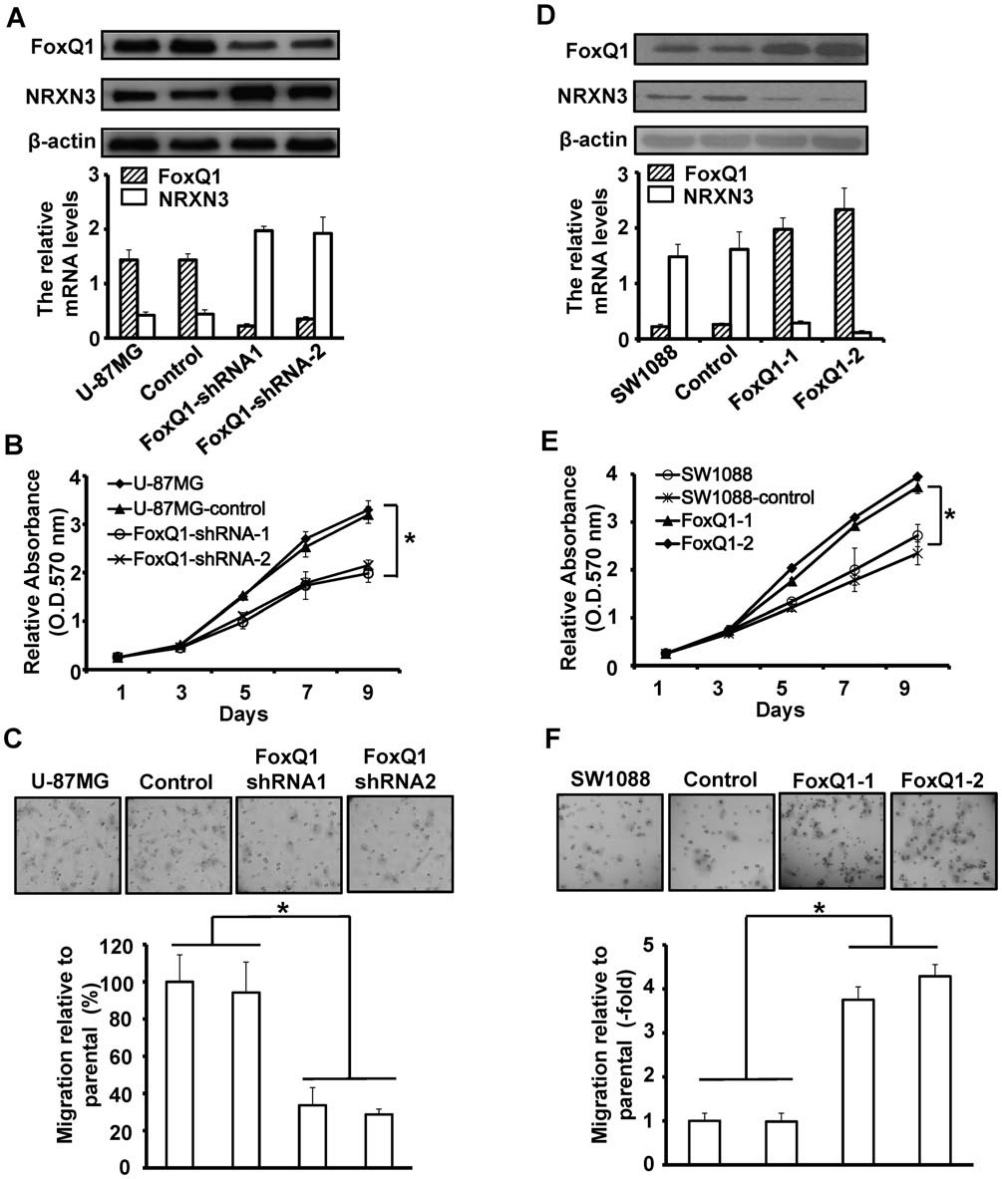
Effect of FoxQ1/NRXN3 expression on proliferation and migration of glioma cells *in vitro*. **A+D**, Western blot (upper) and RT-qPCR (lower) analyses of FoxQ1 and NRXN3 expression in stable FoxQ1shRNA-transfected U-87MG cells (A) and pcDNA3.1-FoxQ1-transfected SW1088 cells (D). **B+E**, Cells as in (A) or (B) were cultured in 96-well plates and analyzed by MTT assay. Cell proliferation curves were shown in 9 days. Three independent experiments were conducted. **C+F**, Cells as in (A) or (B) were examined for cell migration motility in 24-well plates with transwell chambers. Migrated cells were stained with crystal violet and counted under a light microscope. Three independent experiments were conducted. *P<0.05.

Next, we tested the function of FoxQ1/NRXN3 interaction by assessing their roles in glioma cells biological behaviors. We established two stable clones FoxQ1-shRNA-transfected U-87MG (FoxQ1-shRNA-1 and FoxQ1-shRNA-2) as well as vector control clones. RT-qPCR and Western blot analyses showed that the FoxQ1 mRNA and protein levels were decreased in FoxQ1-shRNA-1 and FoxQ1-shRNA-2, whereas the cells showed increased NRXN3 mRNA and protein levels in FoxQ1-shRNA-1 and FoxQ1-shRNA-2 (Fig. 4A). To observe the effects of FoxQ1/NRXN3 on the glioma cells, cell proliferation in FoxQ1-shRNA stable clones were evaluated by MTT assay. During 9-day observations, we found that the proliferation rate of FoxQ1-shRNA cells was apparently lower compared to the control and parental cells (Fig. 4B). Furthermore, we performed transwell assay to assess the function role of FoxQ1/NRXN3 in glioma cells migration. The rate of migrated cells was lower in FoxQ1-shRNA cells than the control and parental cells (Fig. 4C).

Conversely, to determine the effect of increased FoxQ1 expression on NRXN3 expression, we established another two stable clones pcDNA3.1-FoxQ1-transfected SW1088 cells (FoxQ1-1 and FoxQ1-2) as well as their vector control. RT-qPCR and Western blot analyses showed that the FoxQ1 mRNA and protein levels were increased in FoxQ1-1 and FoxQ1-2, whereas the cells showed decreased NRXN3 mRNA and protein levels (Fig. 4D). MTT assay showed that proliferation rate of FoxQ1-1/2 cells was apparently higher compared to the control and parental cells (Fig. 4E). Moreover, Transwell assay showed that the rate of migrated cells was higher in FoxQ1-1/2 cells than the control and parental cells (Fig. 4F).

Together, our results indicated that FoxQ1 enhances the ability of glioma cells proliferation and migration by down-regulation of NRXN3 expression *in vitro*.

### Down-regulation of NRXN3 rescues the malignant phenotype of FoxQ1 down-regulated glioma cells *in vitro* and *in vivo*

**Figure 5:**
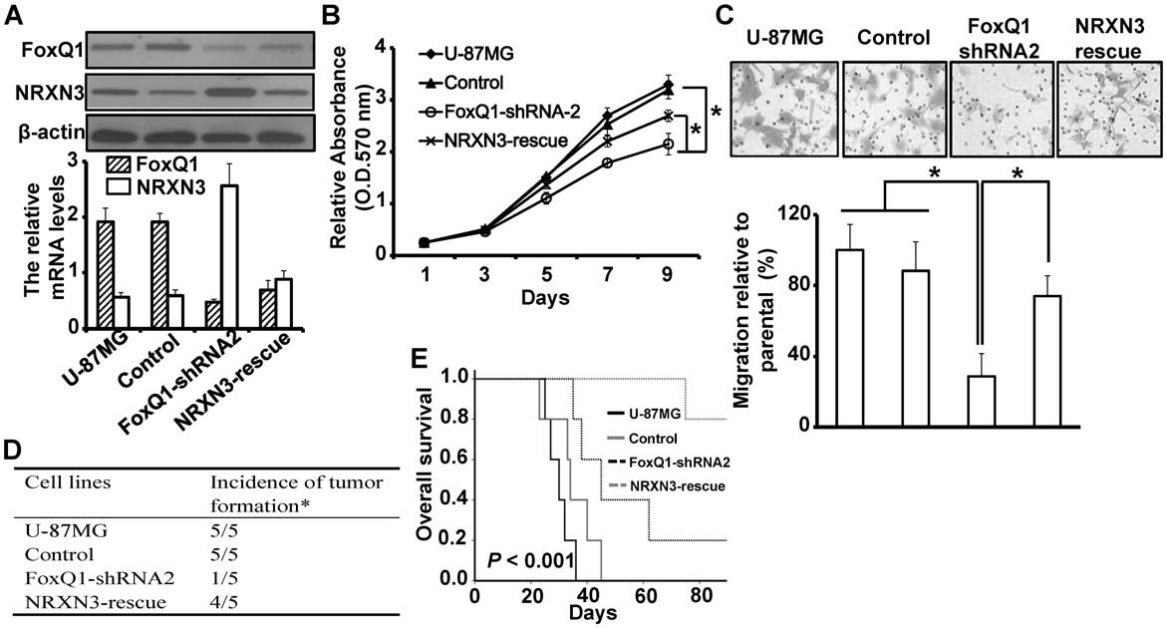
Down-regulation of NRXN3 rescues the malignant phenotype of FoxQ1 down-regulated glioma cells *in vitro* and *in vivo*. A, Western blot (upper) and RT-qPCR (lower) analyses of FoxQ1 and NRXN3 expression in stable NRXN3-rescued U-87MG cells. B, Cells as in (A) were cultured in 96-well plates and analyzed by MTT assay. Cell proliferation curves were shown in 9 days. Three independent experiments were conducted. C, Cells as in (A) were examined for cell migration motility in 24-well plates with transwell chambers. Migrated cells were stained with crystal violet and counted under a light microscope. Three independent experiments were conducted. D, Glioma cells (1×10^6^) were implanted intracranially into nude mice. Mice were euthanized when they were moribund or on day 90. * Incidence: number of mice with tumor/number of mice injected. E, Kaplan-Meier estimates of overall survival time in nude injected with glioma cells. *P<0.05.

To provide direct evidence that FoxQ1 affect the malignant phenotype by down-regulation of NRXN3 in glioma cells, we transfected NRXN3 shRNA into FoxQ1-shRNA2 to rescue the NRXN3 expression and established the stable clone (NRXN3-rescue). RT-qPCR and Western blot analyses showed that the mRNA and protein levels of NRXN3 were down-regulated in NRXN3-rescue cells (Fig. 5A). To observe the effects of FoxQ1/NRXN3 regulation on the glioma cells, cell proliferation and migration in stable clones were evaluated by MTT and migration assay. As shown in Fig. 5B and C, FoxQ1-dependent stimulation of cell proliferation and migration was rescued by down-regulation of NRXN3 in NRXN3-rescue cells.

All the results described above pointed out a possibility that FoxQ1 promote glioma progression by regulating NRXN3. To test this possibility, we intracranially injected U-87MG, U-87MG-control, FoxQ1-shRNA2, and NRXN3-rescue cells into nude mice and found that U-87MG and Control cells produced brain tumors in all of the injected mice, and NRXN3-rescue cells produced brain tumor in 4 of 5 injected mice, however, the FoxQ1-shRNA2 cells produced brain tumor in only one injected mice (Fig. 5D). Furthermore, the mice became moribund ∼40 days after the injection. In contrast, the mice injected FoxQ1-shRNA2 cells have a significant increase in overall survival time (Fig. 5E; *P*<0.001). Our results indicate that inhibition of FoxQ1 expression significantly suppresses the tumorigenicity of human glioblastoma cells, whereas rescue the NRXN3 expression can recover the ability of tumorigenicity.

## Discussion

Fox transcription factors, an evolutionarily conserved superfamily, control a wide spectrum of biological processes. Several Fox gene family members are involved in carcinogenesis and thought to act as either an oncogene or a tumor suppressor [5], [25], [26]. Although previous studies have suggested that FoxQ1 plays an important role in the tumorigenesis of several malignancies, including non-small cell lung cancer, breast cancer, and colorectal cancer [13]–[15], [27], its role and molecular mechanisms in glioma are not known.

FoxQ1 belongs to the human Forkhead-Box (Fox) gene family, which consists of at least 43 members. Deregulation of the Fox family genes, caused by various mechanisms such as amplification, mutation, and gene fusion, leads to congenital disorders, diabetes mellitus, even carcinogenesis [28]. Many Fox family genes play key roles in vertebrate development. Specifically, FoxQ1 has been shown to be a downstream mediator of Hoxa1 in embryonic stem cells [29]. Moreover, overexpression of FoxQ1 has been reported in several cancers including lung cancer [27], [30], pancreatic ductal adenocarcinomas [31], colorectal cancer [13] and breast cancer [2]. Consistent with these results, we found that the FoxQ1 expression level was higher in glioma specimens, whereas the NRXN3 expression level was higher in normal brain tissues. FoxQ1 expression negatively related to the NRXN3 expression in glioma specimens. Moreover, we found that FoxQ1 suppresses NRXN3 through direct binding to the *NRXN3* gene promoter. Inhibition of FoxQ1 in glioma cells by transfection of FoxQ1 shRNA significantly up-regulated NRXN3 expression and reduced the ability of proliferation and migration in glioma cells, whereas overexpression of a FoxQ1 expression vector did the opposite. Therefore, FoxQ1 overexpression contributes directly to NRXN3 underexpression in gliomas and seems to be critical for glioma development.

In this study, we found both clinical and causal experimental evidence that aberrant FoxQ1 expression critically regulates the tumorigenicity of human glioma cells. We sought to determine the molecular mechanism by which FoxQ1 promote glioma development by down-regulating NRXN3 expression. Our RT-qPCR analyses showed a significant association between FoxQ1 overexpression and decreased NRXN3 expression in 30 matched primary glioblastoma tissues and the adjacent normal brain tissues and Western blot further confirmed the correlation in 6 matched specimens. Our findings suggest that FoxQ1 could be a critical pathway in glioma tumorigenesis, which is supported by a recent report showing that FoxQ1 is overexpressed in colorectal cancer [13]. Moreover, to our knowledge, this is the first report to show that NRXN3 is a direct target of FoxQ1. Specifically, we identified two FoxQ1 binding sites in NRXN3 promoter region. FoxQ1 seemed to crucially regulate NRXN3 expression through direct interaction with NRXN3 promoter, as mutation of FoxQ1 binding sites significantly up-regulated NRXN3 promoter activity in glioma cells. Finally, the FoxQ1 expression levels directly affected the glioma cells proliferation and migration in a NRXN3-dependent manner both *in vitro* and *in vivo*. Thus, our work indicated that FoxQ1 regulates gliomas development by down-regulation of NRXN3 expression.

Recently, accumulating evidence has shown that FoxQ1 to be a valuable prognostic indicator for poor outcome in patients with breast cancer and non-small cell lung cancer. Furthermore, high expression of FoxQ1 was also observed in lung cancer, gastric cancer, and colon cancer cell lines [13]. Our present results indicated that FoxQ1 was also high expression in gliomas. Furthermore, we first identified NRXN3 was down-regulation in the glioma specimens, which suggested that NRXN3 is a potential tumor suppressor. The relation between FoxQ1/NRXN3 expression and survival of patients with gliomas need to be clarified in future study.

In conclusion, we have shown that FoxQ1 was highly expressed, whereas NRXN3 was lowly expressed in gliomas. Of more importance, we found that FoxQ1 directly regulated NRXN3 expression and glioma proliferation and migration. Because of the diverse roles of FoxQ1 in cancer development, including regulation of tumor cell proliferation, invasion, angiogenesis, and anti-apoptosis, a better understanding of FoxQ1 signaling and function may help identify novel and effective targets for cancer therapy.

## References

[1] Jemal A, Siegel R, Xu J, Ward E (2010) Cancer statistics, 2010. CA Cancer J Clin 60: 277–300. 20610543 10.3322/caac.20073

[2] Paulino VM, Yang Z, Kloss J, Ennis MJ, Armstrong BA, et al (2010) TROY (TNFRSF19) is overexpressed in advanced glial tumors and promotes glioblastoma cell invasion via Pyk2-Rac1 signaling. Mol Cancer Res 8: 1558–1567. 20881009 10.1158/1541-7786.MCR-10-0334 PMC3092528

[3] Jonsson H, Peng SL (2005) Forkhead transcription factors in immunology. Cell Mol Life Sci 62: 397–409. 15719167 10.1007/s00018-004-4365-8 PMC11924438

[4] Carlsson P, Mahlapuu M (2002) Forkhead transcription factors: key players in development and metabolism. Dev Biol 250: 1–23. 12297093 10.1006/dbio.2002.0780

[5] Myatt SS, Lam EW (2007) The emerging roles of forkhead box (Fox) proteins in cancer. Nat Rev Cancer 7: 847–859. 17943136 10.1038/nrc2223

[6] Ye H, Kelly TF, Samadani U, Lim L, Rubio S, et al (1997) Hepatocyte nuclear factor 3/fork head homolog 11 is expressed in proliferating epithelial and mesenchymal cells of embryonic and adult tissues. Mol Cell Biol 17: 1626–1641. 9032290 10.1128/mcb.17.3.1626 PMC231888

[7] Korver W, Roose J, Clevers H (1997) The winged-helix transcription factor Trident is expressed in cycling cells. Nucleic Acids Res 25: 1715–1719. 9108152 10.1093/nar/25.9.1715 PMC146663

[8] Laoukili J, Kooistra MR, Bras A, Kauw J, Kerkhoven RM, et al (2005) FoxM1 is required for execution of the mitotic programme and chromosome stability. Nat Cell Biol 7: 126–136. 15654331 10.1038/ncb1217

[9] Wonsey DR, Follettie MT (2005) Loss of the forkhead transcription factor FoxM1 causes centrosome amplification and mitotic catastrophe. Cancer Res 65: 5181–5189. 15958562 10.1158/0008-5472.CAN-04-4059

[10] Potter CS, Peterson RL, Barth JL, Pruett ND, Jacobs DF, et al (2006) Evidence that the satin hair mutant gene Foxq1 is among multiple and functionally diverse regulatory targets for Hoxc13 during hair follicle differentiation. J Biol Chem 281: 29245–29255. 16835220 10.1074/jbc.M603646200

[11] Hong HK, Noveroske JK, Headon DJ, Liu T, Sy MS, et al (2001) The winged helix/forkhead transcription factor Foxq1 regulates differentiation of hair in satin mice. Genesis 29: 163–171. 11309849 10.1002/gene.1020

[12] Hoggatt AM, Kriegel AM, Smith AF, Herring BP (2000) Hepatocyte nuclear factor-3 homologue 1 (HFH-1) represses transcription of smooth muscle-specific genes. J Biol Chem 275: 31162–31170. 10896677 10.1074/jbc.M005595200

[13] Kaneda H, Arao T, Tanaka K, Tamura D, Aomatsu K, et al (2010) FOXQ1 is overexpressed in colorectal cancer and enhances tumorigenicity and tumor growth. Cancer Res 70: 2053–2063. 20145154 10.1158/0008-5472.CAN-09-2161

[14] Qiao Y, Jiang X, Lee ST, Karuturi RK, Hooi SC, et al (2011) FOXQ1 regulates epithelial-mesenchymal transition in human cancers. Cancer Res 71: 3076–3086. 21346143 10.1158/0008-5472.CAN-10-2787

[15] Zhang H, Meng F, Liu G, Zhang B, Zhu J, et al (2011) Forkhead transcription factor foxq1 promotes epithelial-mesenchymal transition and breast cancer metastasis. Cancer Res 71: 1292–1301. 21285253 10.1158/0008-5472.CAN-10-2825 PMC3906209

[16] Ushkaryov YA, Petrenko AG, Geppert M, Sudhof TC (1992) Neurexins: synaptic cell surface proteins related to the alpha-latrotoxin receptor and laminin. Science 257: 50–56. 1621094 10.1126/science.1621094

[17] Ullrich B, Ushkaryov YA, Sudhof TC (1995) Cartography of neurexins: more than 1000 isoforms generated by alternative splicing and expressed in distinct subsets of neurons. Neuron 14: 497–507. 7695896 10.1016/0896-6273(95)90306-2

[18] Occhi G, Rampazzo A, Beffagna G, Antonio Danieli G (2002) Identification and characterization of heart-specific splicing of human neurexin 3 mRNA (NRXN3). Biochem Biophys Res Commun 298: 151–155. 12379233 10.1016/s0006-291x(02)02403-8

[19] Rowen L, Young J, Birditt B, Kaur A, Madan A, et al (2002) Analysis of the human neurexin genes: alternative splicing and the generation of protein diversity. Genomics 79: 587–597. 11944992 10.1006/geno.2002.6734

[20] Ushkaryov YA, Sudhof TC (1993) Neurexin III alpha: extensive alternative splicing generates membrane-bound and soluble forms. Proc Natl Acad Sci U S A 90: 6410–6414. 8341647 10.1073/pnas.90.14.6410 PMC46941

[21] Tabuchi K, Sudhof TC (2002) Structure and evolution of neurexin genes: insight into the mechanism of alternative splicing. Genomics 79: 849–859. 12036300 10.1006/geno.2002.6780

[22] Kusinska R, Gorniak P, Pastorczak A, Fendler W, Potemski P, et al (2012) Influence of genomic variation in FTO at 16q12.2, MC4R at 18q22 and NRXN3 at 14q31 genes on breast cancer risk. Mol Biol Rep 39: 2915–2919. 21688152 10.1007/s11033-011-1053-2 PMC3271204

[23] Hishimoto A, Liu QR, Drgon T, Pletnikova O, Walther D, et al (2007) Neurexin 3 polymorphisms are associated with alcohol dependence and altered expression of specific isoforms. Hum Mol Genet 16: 2880–2891. 17804423 10.1093/hmg/ddm247

[24] Marinescu VD, Kohane IS, Riva A (2005) The MAPPER database: a multi-genome catalog of putative transcription factor binding sites. Nucleic Acids Res 33: D91–97. 15608292 10.1093/nar/gki103 PMC540057

[25] Paik JH, Kollipara R, Chu G, Ji H, Xiao Y, et al (2007) FoxOs are lineage-restricted redundant tumor suppressors and regulate endothelial cell homeostasis. Cell 128: 309–323. 17254969 10.1016/j.cell.2006.12.029 PMC1855089

[26] Kalin TV, Wang IC, Ackerson TJ, Major ML, Detrisac CJ, et al (2006) Increased levels of the FoxM1 transcription factor accelerate development and progression of prostate carcinomas in both TRAMP and LADY transgenic mice. Cancer Res 66: 1712–1720. 16452231 10.1158/0008-5472.CAN-05-3138 PMC1363687

[27] Feng J, Zhang X, Zhu H, Wang X, Ni S, et al (2012) FoxQ1 Overexpression Influences Poor Prognosis in Non-Small Cell Lung Cancer, Associates with the Phenomenon of EMT. PLoS One 7: e39937. 22761930 10.1371/journal.pone.0039937 PMC3386178

[28] Katoh M (2004) Human FOX gene family (Review). Int J Oncol 25: 1495–1500. 15492844

[29] Martinez-Ceballos E, Chambon P, Gudas LJ (2005) Differences in gene expression between wild type and Hoxa1 knockout embryonic stem cells after retinoic acid treatment or leukemia inhibitory factor (LIF) removal. J Biol Chem 280: 16484–16498. 15722554 10.1074/jbc.M414397200

[30] Bieller A, Pasche B, Frank S, Glaser B, Kunz J, et al (2001) Isolation and characterization of the human forkhead gene FOXQ1. DNA Cell Biol 20: 555–561. 11747606 10.1089/104454901317094963

[31] Cao D, Hustinx SR, Sui G, Bala P, Sato N, et al. (2004) Identification of novel highly expressed genes in pancreatic ductal adenocarcinomas through a bioinformatics analysis of expressed sequence tags. Cancer Biol Ther 3: : 1081–1089; discussion 1090–1081. 10.4161/cbt.3.11.1175 15467436

